# Knowledge, Attitude and Practice of Nurses about Standard Precautions for Hospital-Acquired Infection in Teaching Hospitals Affiliated to Zabol University of Medical Sciences (2014)

**DOI:** 10.5539/gjhs.v8n3p193

**Published:** 2015-07-27

**Authors:** Hamed Sarani, Abbas Balouchi, Nosratollah Masinaeinezhad, Ebrahim Ebrahimitabs

**Affiliations:** 1 Pregnancy Health Research Center, Zahedan University of Medical Sciences, Zahedan, IR Iran; 2 Research Committee Center, School of Nursing and Midwifery, Zabol University of Medical Sciences, Zabol, IR Iran; 3 School of Nursing and Midwifery, Zabol University of Medical Sciences, Zabol, IR Iran

**Keywords:** knowledge, attitude, practice, standard precautions, infection control

## Abstract

**Background and Objectives:**

Hospital-acquired infection (HAI) is one of the common problems and difficulties faced by hospitals in all countries around the world. Since nurses are part of the healthcare team that plays a unique role in the control of hospital infection, this study is conducted to analyze the knowledge and practice of healthcare personnel about standard precautions for hospital infection.

**Materials and Methods:**

This descriptive study was conducted on 170 nurses worked in medical surgical wards, pediatric wards, dialysis units of two teaching hospitals in Zabol city, Iran, in 2014. The sample population was selected through simple random sampling. The data collection instrument is composed of a researcher-made questionnaire titled “Hospital-acquired infection Control” based on precautions posited by the World Health Organization (WHO) and the United States Centers for Disease Control and Prevention (CDC). Data were fed into the SPSS software v.20 and were analyzed using descriptive and inferential statistics.

**Results:**

The results show that 43% of the participants in this study had poor knowledge, 42% had average practice, and 37% had a moderate attitude about hospital infection. There was a significant relationship between knowledge and gender (r = 00.8 p = 0.02). However, the variables of age, marital status, employment, work experience, education, and place of work did not establish a significant relationship with the independent variables (p>0.05).

**Conclusion:**

As the results indicate a low level of awareness among the personnel about hospital infection, it is suggested to provide training sessions on the prevention and control of HAI to increase the awareness of personnel and hold practical courses for practicing these principles.

## 1. Introduction:

HAI is a major health problem in all societies. According to the WHO, 7.1 million cases of HAI occur every year. One out of every 20 people suffers from hospital infection. This leads to 99,000 cases of death every year and imposes an estimated cost of $ 32 million to society (Cardo et al., 2010). On arrival at the hospital, patients do not have HAI but they may develop it during the 72 hours or more after hospitalization (Horan et al., 1992). The WHO has provided a general definition of HAI and has renamed it as healthcare-associated infection. WHO launched its activities in 2005 under the slogan, “clean care is safer care”. HAI is an infection that develops in a limited or vast scope because of pathogenic reactions associated with the infectious agent or its toxins in the hospital (but only if the infection is caused at least during the 48 to 72 hours after admission to the hospital or during a specified period of 3 to 10 days after dismissal. So the patient must not show symptoms of infection at the time of admission and the infection must not be in the incubation period (Boyce & Pittet, 2002; Safari & Shojaei, 2002). A study conducted by the WHO on 55 hospitals in 14 countries showed that 8.7% of the patients hospitalized in these hospitals became infected with healthcare-associated infection. Studies conducted in Iran on the incidence of HAI and the subsequent increased length of stay and costs show that HAI is the most important socioeconomic medical problem in the country. Simutaneousley, through hospital treatment for acute diseses, people with long-term diseases will cure too and hospital treatment will become more sophisticated and therefore hospital stay will be longer which this leads in to HAI (Arbabisarjou, 2012). The incidence HAI in Iran has been reported to vary on a range from 1.9% to more than 25% (Angelillo et al., 1999).

Numerous factors are associated with high risk of HAI. The factors that can minimize the risk of HAI include the systematic treatment of patients, avoiding prolonged hospitalization, the use of antibiotics, the use of suction catheters, hand washing by health care personnel, and the use of sterilization techniques in therapeutic procedures (Ayliffe et al., 2000). The high costs of treating large numbers of patients and increasing occurrences of infection have posed a threat to standard precautions because these standards constitute the basic principles of HAI control. HAI control means the reduction of infection risk by patients, hospital personnel, and patient care attendants and the prevention of infection transmission by hospital personnel and patients’ families (Amerioun et al., 2009). As members of the health care team, nurses play a very important role in HAI control (Saffari et al. 2008). Nurses must have sufficient information and necessary skills in this field (Saleh Moghadam, 2005). The results of a study conducted by Darawad et al. on nursing students in Yemen showed that most nursing students have low levels of knowledge, a positive attitude, and a moderate practice about infection control (Darawad & Al-Hussami, 2013). A study by Hinkin et al. showed that most students have acceptable levels of knowledge about infections, hand hygiene, the use of gloves and taking appropriate action after being injured by a sharp object but had low levels of knowledge about the use of gel and other disinfecting procedures. The results also showed that their level of knowledge depended on working pressure, time and access to facilities (Hinkin & Cutter, 2014). Ghanbari et al. conducted a study on 130 nurses. The results showed that most nurses do not have sufficient knowledge and practice about the prevention of hospital infection (Ghanbari et al., 2013). The observation of health procedures is therefore the most fundamental health principle and the most basic health behavior (Mac, 2002). The prevention of HAI requires attention to three concepts: knowledge, attitude, and practice (Saffari & Shojaei, 2002). For the occurrence of a behavior, the presence of such factors as motivation and emotion is necessary. This study was conducted to evaluate knowledge, attitude and practice of nurses against HAI.

## 2. Materials and Methods

The present study was conducted using a descriptive cross-sectional on 170 nurses working at teaching hospitals of Zabol, Iran, under the supervision of Zabol University of Medical Sciences in 2014. The sample population was selected through simple random sampling. Inclusion criteria were having at least a bachelor's degree and a work experience of at least three months. The only exclusion criterion was reluctance to participate in the study. The location of study covered internal medicine wards, Pediatrics wards, dialysis units, and surgical units of Amir al-Momenin hospital and Imam Khmoeini hospital in Zabol city, Iran. For data collection, it used a researcher-made questionnaire with confirmed validity and reliability by several preceding studies. The questionnaire consists of two main parts: the first part collects demographic information including gender, age, work experience, ward of activity, and a history of infection control training; the second part consists of three subsections: the first subsection, Knowledge, including 5 items about the nature of infection, mode of transmission, prevention of infection and the role of the nurse; the second subsection, practice, including 21 items measuring individual practice in relation to the adoption of preventive behaviors, hand washing, injection and dressing skills, and the observation of precautions; the third subsection, attitude, including 10 items measuring perceived threat by nurses about HAI of nurses and patients and perceived benefits by patients about the observation of standard precautions. In the Knowledge subsection, each correct answer to the items was scored 1 and each wrong answer was scored zero. In the end, the scores were calculated in percent - the number of correct answers multiplied by 100 divided by the total number of items. The practice subsection was comprised of 10 items rated on a 5-point Likert scale from 1 to 5 (Strongly Disagree=1, Disagree=2, Neutral=3, Agree=4, Strongly Agree= 5). The total scores ranged between 1 and 20 and the individual scores for each section were calculated in percent. Items in the practice checklist were rated on the Likert scale. Individual practice scores were calculated based on the frequency of adopting preventive behaviors against HAI (according to standard precautions) divided by the total number of listed behaviors multiplied by 100. Items in the attitude subsection were similarly rated on a five-point Likert scale. In analyzing the data in both sections of knowledge and practice of nurses in the context of standard precautions, the scores of nurses were categorized as low, medium, and good. Scores below 50 were labeled as poor, scores between 50 and 75 were labeled as average, and scores above 75 were considered as good. The study was conducted after obtaining informed consent from nurses. Data were collected via questionnaires and were analyzed with the SPSS software product v.20 using descriptive statistics and correlation coefficient Pearson correlation test. Level of significance P value is ≤0.05 regarded as statistically significant.

## 3. Results

Of the 170 questionnaires distributed, 145 completed questionnaires were selected for analysis. The mean age of participants was equal to 41±1.13, and the mean duration of employment was 8± 2.1 years. The mean score of the participants on the knowledge of infection was poor (42.5±8). The highest levels of knowledge were related to hand hygiene with a mean of 74.5±24 and precautions to avoid needle stick injuries with a mean of 70±3. In addition, the lowest level of knowledge was related to precautions such as wearing the gown, gloves, mask and glasses during clinical procedures with a mean of 64±2.8. Of the 145 nurse participants in this study, 43% (n=63) had poor knowledge, 35% (n=51) had average knowledge, and 22% (n=31) of the nurses had good knowledge about the prevention of HAIs. Based on the Pearson correlation coefficient, there is no statistically significant relationship between knowledge and practice (r=00.8 p=0.3). However, there is a significant relationship between knowledge and gender (p = 0.02). Besides, the variables of age, marital status, employment, work experience, education, and place of work did not establish a significant relationship with the independent variables (p>0.05). Out of 145 participants in this study, 24% (n=34) of the nurses had poor practice, 42% (n=61) had average practice, and 34% (n=50) had good practice in the prevention of HAIs (Table 1). No statistically significant association was observed between knowledge and practice (p<0.05).

**Table 1:** Knowledge, attitude and practice of nurses about infection control standards

Variable	Level	Frequency (no.)	Percent (%)	Mean ± SD
**Knowledge**	Poor	**63**	**43**	42±8.9
	Average	**51**	**35**	
	Good	**31**	**22**	
**Practice**	Poor	**34**	**24**	48±7.5
	Average	**61**	**42**	
	Good	**50**	**34**	
**Attitude**	Poor	**43**	**30**	40±6.2
	Average	**54**	**37**	
	Good	**48**	**33**	

In relation to the attitude of nurses, the results showed that 30% (n=43) of nurses had a poor attitude, 37% (n=54) had an average attitude, and 33% (n=33) had a good attitude about infection control. A statistically significant relationship was found between the mean scores of nurses who had been trained and those who had not passed training courses (p<0.01). The results also showed that those who had more knowledge about infection control had a better practice. The results of the Pearson correlation coefficient test for the assessment of the relationship between the knowledge, attitude and practice of nurses showed that the attitude of nurses was significantly correlated with their practice (p<0.01 and r=46). The results also indicated that nurses who were less experienced had average levels of knowledge.

## 4. Discussion

The findings of this study showed that most nurses had a poor knowledge (43%), an average practice (42%), and a moderate attitude (37%) about HAI control. The results of this study are not consistent with the results of a study conducted by Yang Luo et al. in China on 1,444 nurses, in which they assessed the knowledge of nurses about standard precautions as average (Luo et al., 2010). In their study on the knowledge, attitude and practice of nurses in the context of HAI control, Ghadamgahi et al. concluded that most nurses do not have a good knowledge of HAI (Ghadmgahi et al., 2011). The results of this study are not consistent with the finding of the study by Gould et al. on 173 nurses working in three wards (ICU, Medical-surgical wards), in which they assessed the knowledge of nurses about standard precautions as low (Gould & Chamberlain, 1994). The results of a study by D’Alessandro et al. showed that 90.8% of students had a poor knowledge about infection control (D’Alessandro et al., 2014). The results of another study by Sodhi et al. showed that more than 90% of ICU nurses had a very good knowledge of infection control (Sodhi et al., 2013). Chan's study also showed that 56% of nurses had a good knowledge about infection control and 79% of them had a good practice in relation to standard precautions for infection control (Chan et al., 2002). Allah-Bakhshian et al. assessed the knowledge, attitude and practice of ICU nurses working at training centers in Tabriz, Iran, about hospital infection control and concluded that the majority of nurses in this study had an average knowledge about HAI control (Allah-Bakhshian et al., 2010). It is important to note that the knowledge of nurses about HAI depends on many factors, including individual and educational characteristics, training courses, and managerial and motivational factors. In their study on the knowledge, attitude and practice of different groups of healthcare personnel about infection control, Suchitra et al. concluded that training has a positive impact on the improvement of knowledge, attitude and practice in healthcare personnel. They also suggested that the development of a continuous training program for all healthcare workers is necessary (Suchitra, 2007). Training courses have been shown to be effective in promoting the knowledge and practice of health care personnel in the UK (Elliott et al., 2005). Training and knowledge improvement are the most effective ways to fight HAI. Obviously, continuous training and knowledge improvement besides the use of appropriate and effective methods of disinfection and sterilization will reduce the frequency of developing HAI (Askarian et al., 2004). The results of a study by Nasirudeen et al. on the knowledge and practice of students in Singapore showed that 66.3% of them had a have good practice and 48.9% of them had a good knowledge about hand hygiene (Nasirudeen et al., 2012). It seems that since infection control topics are not included in academic nursing courses and since they are not dealt with in the work environment either, nurses have a poor knowledge in this area. Therefore, considering the guidelines on the treatment of hospital infection - that nurses should be trained and retrained at least twice a year (Bischoff et al., 2000)- differences in the results can be interpreted. There was a significant relationship between knowledge and gender which is consistent with the results of the study (Ghadmgahi et al., 2011). The results of the present study showed that nurses have a poor practice in the prevention of HAIs (Akyol, 2007). Bischoff et al. claimed that under normal conditions, the frequency of hand washing by doctors and nurses was at an unacceptably low level. A study in India reported less than desirable levels of practice among healthcare personnel (Bischoff et al., 2000). Akyol (2007) noted that hand hygiene compliance by healthcare workers was at a poor level. This is not consistent with the results of the study by Allah-Bakhshian in which almost all participants (99.1%) had an average practice in relation to infection control (Allah-Bakhshian et al., 2010). A study in Jamaica showed that 85% of nurses, despite having the knowledge, did not observe all safety precautions when performing nursing procedures (Figueroa et al., 1997). Mahmoudi and Hassani (2000) stated that the mere having of knowledge does not lead to good practice so attitudes should also change and belief structures should be reworked in a rigorous and scientific manner to achieve proper practice. There was a significant relationship between knowledge and practice in the present study. The study by Lou also reported a significant relationship between knowledge and practice(Luo et al., 2010). One of the limitations of this study is that the sample population does not represent all nurses in Iran.

## 5. Conclusion

According to the results, most nurses do not have a good knowledge and practice about infection control despite having an average efficacy. Therefore, it is necessary that Iran's Ministry of Health and Medical Education and the subsidiary universities do their best to inform the nurses and all the medical personnel about the prevention of HAIs according to world standards and tailored to each region's ecology by way of academic courses, posters, and conferences. It is also necessary to improve the knowledge of standard precautions, develop programs for HAI control, and hold training courses based on successful educational models.
